# Sleep patterns in Amazon rubber tappers with and without electric light at home

**DOI:** 10.1038/srep14074

**Published:** 2015-09-11

**Authors:** C. R. C. Moreno, S. Vasconcelos, E. C. Marqueze, A. Lowden, B. Middleton, F. M. Fischer, F. M. Louzada, D. J. Skene

**Affiliations:** 1 School of Public Health, University of São Paulo, Brazil; 2 Stress Research Institute, Stockholm University, Sweden; 3 Faculty of Health & Medical Sciences, University of Surrey, UK; 4 Department of Physiology, Federal University of Paraná, Brazil; 5 Catholic University of Santos, Brazil

## Abstract

Today’s modern society is exposed to artificial electric lighting in addition to the natural light-dark cycle. Studies assessing the impact of electric light exposure on sleep and its relation to work hours are rare due to the ubiquitous presence of electricity. Here we report a unique study conducted in two phases in a homogenous group of rubber tappers living and working in a remote area of the Amazon forest, comparing those living without electric light (n = 243 in first phase; n = 25 in second phase) to those with electric light at home (n = 97 in first phase; n = 17 in second phase). Questionnaire data (Phase 1) revealed that rubber tappers with availability of electric light had significantly shorter sleep on work days (30 min/day less) than those without electric light. Analysis of the data from the Phase 2 sample showed a significant delay in the timing of melatonin onset in workers with electric light compared to those without electric light (p < 0.01). Electric lighting delayed sleep onset and reduced sleep duration during the work week and appears to interfere with alignment of the circadian timing system to the natural light/dark cycle.

Today there is no doubt of the importance of the light/dark cycle in the regulation and synchronization of the circadian timing system. In non-human animals, the phase resetting effects of light have been demonstrated to be mediated by intrinsically photoreceptive retinal ganglion cells (ipRGCs)^1^,^2^ that express melanopsin, an opsin photopigment^3^,^4^. Laboratory human experiments have shown that light is the primary time cues for entrainment of circadian rhythms, since the absence of light in blind individuals^5^,^6^ or in very dim lighting conditions in sighted people^7^,^8^ leads to free-running non 24-h circadian rhythms. The ability of light to shift circadian timing in humans and the resulting phase response curves to light formed the first key evidence leading to the knowledge we have today^9^,^11^.

Modern society promotes a lifestyle that works against circadian alignment, with increased exposure to artificial light and use of electronic equipment, both only possible due to the advent of electricity. There is accumulating evidence that electronic technology is impacting on our sleep, as has been shown in adolescents^12^,^13^. Light (often blue-enriched) from the use of computers, videogames, television and other electronic devices in the evening may contribute to a delayed sleep onset^14^. On the other hand, adolescents living in rural homes without electric light and electronic equipment were found to have earlier sleep times^15^. Recently, a study of adults camping in tents under natural light/dark conditions followed by a period living in a laboratory with electric lighting showed a change in phase of entrainment, changes in sleep timing as well as reduced exposure to sunlight and widespread use of electric light in the constructed laboratory environment^16^. However, in modern society the balance between artificial and natural light exposure and its influence on people’s health is still unclear. The timing of sleep and daily activities are influenced by a variety of work and social pressures as well as genetic predisposition, thus in order to study the impact of electricity, we selected a relatively isolated homogenous population of rubber tappers living in a remote Amazon Extractive Reserve.

In 2003, the Brazilian government created a program called “Light for everybody”, and formed partnerships to fulfil this commitment. The goal of this governmental program was to install electricity in all houses (including residences, schools, and public places) in the country, that were still without electricity. The Chico Mendes Extractive Reserve located in the Amazon (Brazil) was one of the areas where electricity began to be installed. However, the transport of material to the localities required more than simple logistics, because the houses are very far apart and the paths/access routes are not ideal. Thus, the electrification program is not yet completed, and many residents in the area are still without electricity at home.

Our study thus aimed to compare sleep patterns and daily activity between a group with electric light at home and a group without electric light at home. The study was done in two phases, a questionnaire phase (Phase 1; n = 97 with electric light and n = 243 without electric light), and an objective measures phase (Phase 2; n = 17 with electric light and n = 25 without electric light), where data regarding sleep-wake patterns, melatonin timing and light exposure were collected during work days and days-off.

## Results

### Rubber tappers life style and demographic data

We recruited 340 rubber tappers living in the Chico Mendes Amazon Extractive Reserve, located in the state of Acre, Brazil (latitude 10°39′06″S and longitude 68°30′16″W). Of these, 72% (n = 243) had no electricity at home. The rubber tappers worked outdoors Monday to Friday, from 05:00–06:00 h to 16:00–17:00 h, their task being to cut rubber trees in the Amazon forest to obtain the sap. By sunset these workers had returned to their homes. In this region, the length of the day and night are almost equivalent, resulting in a photoperiod of 12 hours per day throughout the year. Therefore, the sunrise and sunset were very similar in both phases of this study (see Methods for details).

In both groups, the majority of the rubber tappers were males (91.7% electricity vs 91.4% no-electricity, Table 1). The groups also were of similar age (40.5 vs. 42.6 years) and had normal weight (BMI 24.0 vs 23.6 kg/m2) (Table 2). Only civil status was found different between the groups among the demographic variables (Table 1); those having electricity were more often living with a partner (80.4% vs. 70.0%, p = 0.05). The highest educational level reached was most commonly primary school (69.1% vs. 68.3%). Most of the rubber tappers were born within the Amazon Extractive Reserve (81.5% with electricity vs 77% no electricity), thus having a similar photic history of 12 hours of natural light throughout their lifetime (Table 1). Out of 340 workers, there were 251 (73.8%) rubber tappers with children under 15 years old.

Besides participating in the survey (Phase 1), a subgroup of rubber tappers agreed to collect saliva for measurement of melatonin timing and to wear actigraphs and a light sensor for the measurement of objective sleep and light exposure (Phase 2). The volunteers participating in Phase 2 of the study had similar demographics characteristics to those whoparticipated in Phase 1 (Tables 1 and 2).

There were no differences regarding reported sleep quality and awakening problems between those who had electric light at home and those who had not for the Phase 1 sample and the Phase 2 sample (Table 1). Chronotype, however, as measured by the Munich Chronotype Questionnaire, was significantly later for the group with electric light at home in the whole sample (Table 2).

### Sleep duration and timing in Phase 1 – Subjective data

**Figure 1:**
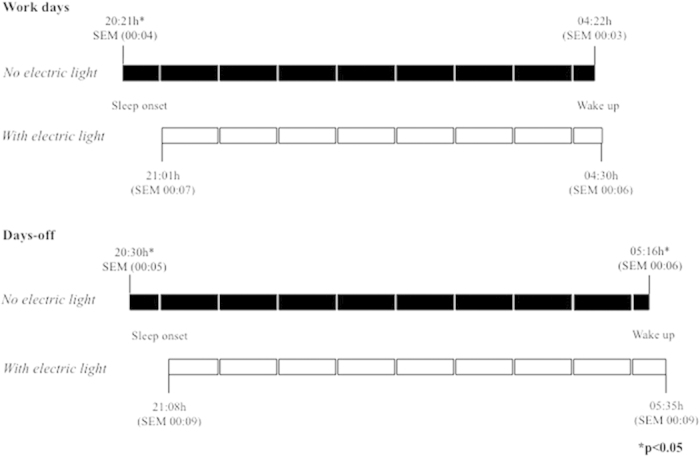
Habitual sleep timing based on questionnaire data (Phase 1) during work days and days-off of rubber tappers with (n = 97) and without (n = 243) electric light at home. *All times indicate the official local time.*

Questionnaire data (subjective data) from the whole sample (n = 340; Phase 1) revealed that workers with electric light at home went to sleep significantly later (21:01 h) than those without electric light (20:21 h, p < 0.01, Fig. 1) during the work week, as well as during days-off (21:08 h and 20:30 h, respectively; p < 0.01). On days-off, workers with electric light also woke up significantly later (05:35 h) than those without electric light (05:16 h) (Fig. 1). Wake up times during the work week were similar for both groups, in agreement with their reported work hours.

Mean subjective sleep duration was 7.5 h (±0.1 h SEM) and 8.4 h (±0.2 h SEM) on work days and days-off, respectively, for the rubber tappers with electric light at home. Workers without electric light at home slept 8.0 h (±0.1 h SEM) and 8.8 h (±0.1 h SEM) during work days and days-off, respectively. Mann-Whitney U test revealed a significant difference in sleep duration between the two groups on work days (p < 0.01), those with electric light at home having significantly shorter sleep (30 min/day less).

### Sleep duration and timing in Phase 2 – Objective data

**Figure 2:**
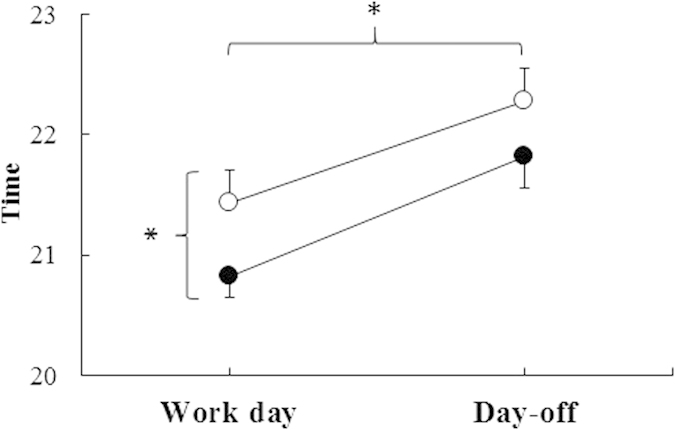
Sleep onset (mean ± SEM) based on actigraphy and sleep diary data (Phase 2) with day of week and availability of electric light as factors (○ with, n = 17, and • without electric light, n = 20). *Electric light F(1, 35)* *=* *4.04, p* *=* *0.05; Day F(1, 35)* *=* *16.93, p* *<* *0.01 and interaction Electric light x Day F(1, 35)* *=* *0.12, p* *=* *0.74. Work day: Monday-Thursday; Day-off: Saturday-Sunday.*

Objective sleep onset on work days was also significantly later for those with (21:26 h, n = 17) compared to those without electric light (20:49 h, n = 20) (p < 0.05) (Fig. 2). We also found a main effect of day of week (p < 0.01), with a delayed sleep onset on days-off. However, there was no interaction between day of week and group (with and without electric light) regarding objective sleep onset times (p = 0.74).

**Figure 3:**
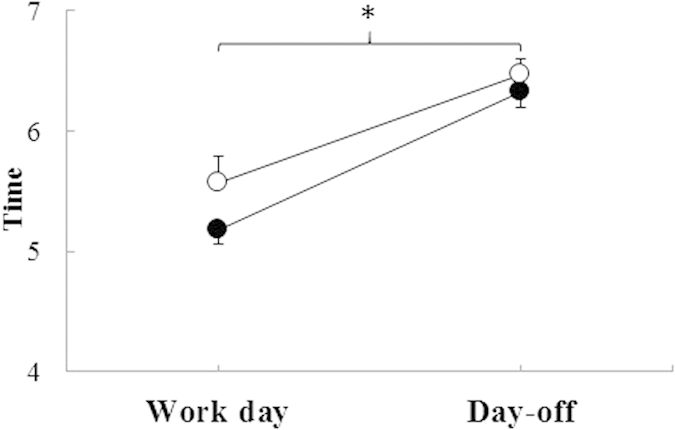
Sleep offset (mean ± SEM) based on actigraphy and sleep diary data (Phase 2) with day of week and availability of electric light as factors (○ with, n = 17, and • without electric light at home, n = 20). *Electric light F(1, 35)* *=* *1.97, p* *=* *0.17; Day F(1, 35)* *=* *70.60, p* *<* *0.01and interaction Electric light x Day F(1, 35)* *=* *1.02, p* *=* *0.32. Work day: Monday-Thursday; Day-off: Saturday-Sunday.*

The group with electric light at home (n = 17) woke up later during the work week than the group without electric light (n = 20), however, this difference was not statistically significant (p = 0.17; Fig. 3). Both groups woke up before sunrise, according to objective measures (Fig. 3). This finding was confirmed by repeated measured ANOVA for wake up times, which found no difference between the groups and no interaction between day of week and groups. However, a main effect of day of week was found, revealing that both groups of rubber tappers woke up later on their days-off (p < 0.01, Fig. 3).

The rubber tappers with electric light at home slept on average 8.0 h (±0.2 h SEM) and 8.2 h (±0.3 h SEM) during the work days and days-off, respectively. Those without electric light slept on average 8.3 h (±0.2 h SEM) on work days and 8.4 h (±0.3 h SEM) on days-off. In Phase 2 of the study the differences in objective sleep duration between the two groups were not statistically significant.

### Melatonin timing in Phase 2

**Figure 4:**
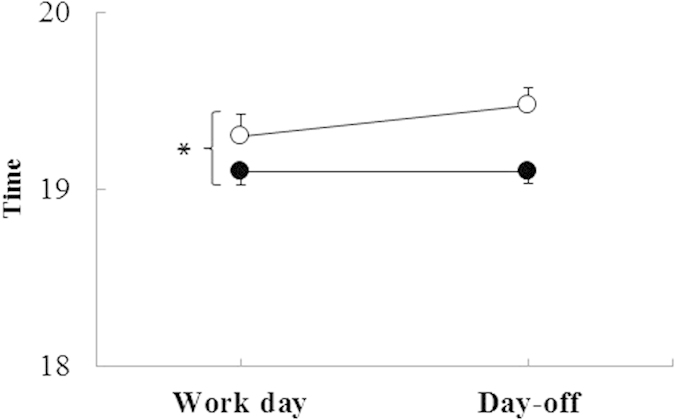
Time of dim light melatonin onset (DLMO) (mean ± SEM) of rubber tappers during one work day and one day-off (○ with, n = 13, and • without electric light, n = 20). *Repeated measures ANOVA with electric light and day of week as factors. Electric light F(1, 31)* *=* *5.38, p* *=* *0.03; Day F(1,31)* *=* *0.69, p* *=* *0.41 and interaction Electric light x Day F(1,31)* *=* *1.31, p* *=* *0.26.*

The timing of the dim light melatonin onset (DLMO) was able to be determined in 33 participants in Phase 2. Analysis showed a significant delay in the timing of melatonin onset in workers with electric light (19:28 h, n = 13) compared to those without electric light (19:06 h, n = 20), revealing a main effect of electric lighting at home (p < 0.01, Fig. 4). On the other hand, there was no day of week effect. The rubber tappers without electric light (n = 20) had the same melatonin onset time during their work day and day-off (19:06 h). Likewise for the rubber tappers with electric light (n = 13), melatonin onset time was not significantly different on days-off (19:47 h) compared to work days (19:28 h). Melatonin onset occurred after sunset in all situations.

Both groups slept later during their days-off than during week days, and do not change melatonin phase during days-off compared to week days. Thus, the time interval (phase angle) between sleep onset and melatonin onset changed between work days and days-off, showing a main effect of day of week (F = 6,13; p = 0.02).

### Activity and light exposure in Phase 2

**Figure 5:**
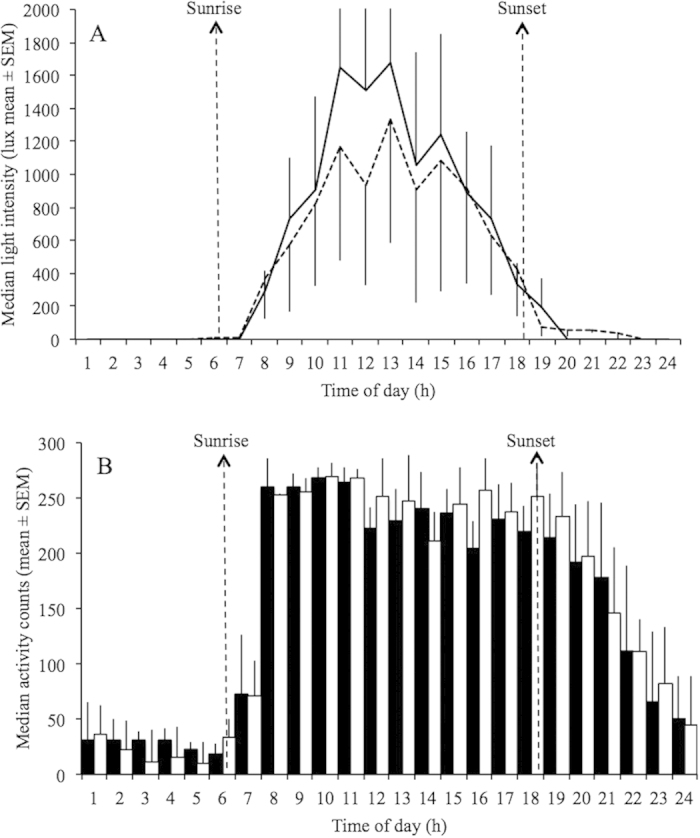
Light levels per hour (A) and activity (B) levels during days-off of rubber tappers with (black lines; white bars; n = 7) and without (dotted lines; black bars; n = 13) electric light at home. *Activity is an arbitrary unit (counting events) and light (lux) was measured by individual neck-worn light sensors. Bars represent activity bins (counts/h) and lines are light intensity (lux). Sunrise and sunset was at 06:48* *h (±0.04* *h SEM) and 18:24 h (±0.04* *h SEM), respectively, according to the official local time.*

**Figure 6:**
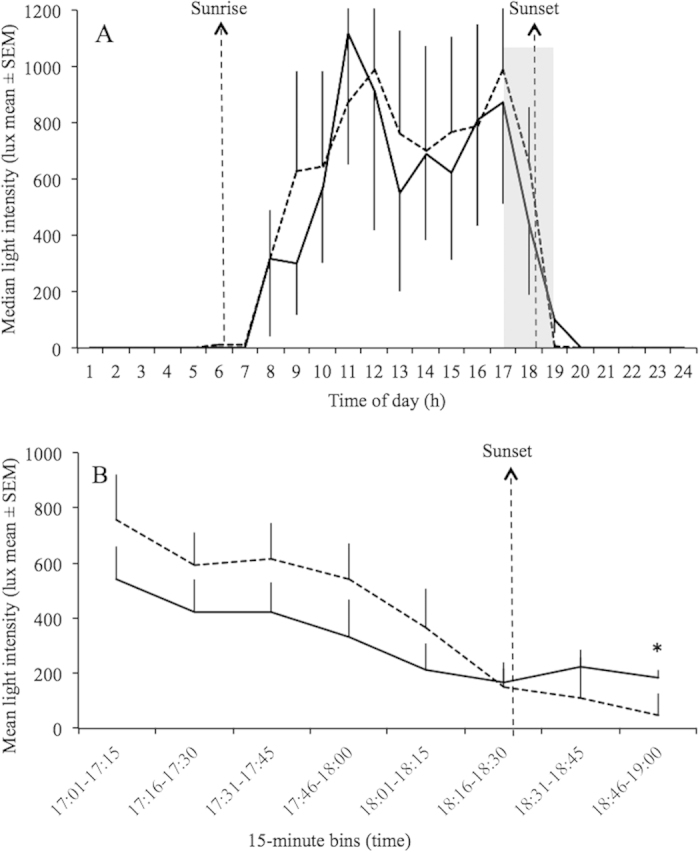
Light levels per hour (A) and 15-min bins from 17:00 to 19:00 h (B) during work week of rubber tappers with (black lines; n = 7) and without (dotted lines; n = 13) electric light at home. *Light (lux) was measured by individual neck-worn light sensors. Sunrise and sunset was at 06:48* *h (±0.04* *h SEM) and 18:24* *h (±0.04* h SEM), respectively, according to the official local time. *Mann-Whitney U test showed a significant difference between groups at 18:46–19:00 *h; p* *<* *0.05.*

The study population was exposed daily to natural light, since all work activity was conducted outdoors. Although all 42 Phase 2 participants wore light and activity monitors, due to humidity and mechanical failure of the sensors, only data from 7 rubber tappers with electric light at home and 13 without electric light were analyzed. In both groups activity was mainly concentrated during daylight hours during the work week and days-off (Figs 5 and 6).

**Figure 7:**
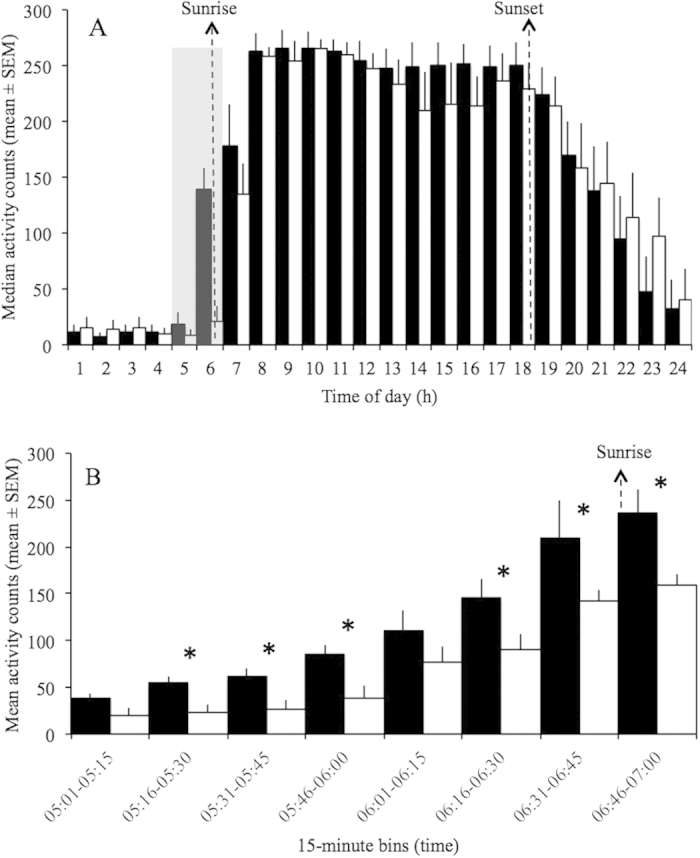
Activity levels per hour (A) and 15-min bins from 05:00 to 07:00 h (B) during work week of rubber tappers with (white bars; n = 7) and without (black bars; n = 13) electric light at home. *Activity is an arbitrary unit (counting events), and bars represent activity bins. Sunrise and sunset was at 06:48* *h (±0.04* *h SEM) and 18:24* h (±0.04 h SEM), respectively, according to the official local time. *Mann-Whitney U test showed a significant difference between groups; p *<* *0.05.*

Light levels (median lux/hour) and activity (median activity counts/hour) were significantly correlated in both groups during the work week (r = 0.85, p < 0.01, and r = 0.84, p < 0.01 for the group with electric light and without electric light, respectively). A similar significant correlation between light and activity levels was found during days-off (r = 0.83, p < 0.01, and r = 0.87, p < 0.01) for the group with electric light and without electric light, respectively). Mann-Whitney U test revealed a significant difference in light exposure between the groups within the interval 18:45–19:00 h (p < 0.05), in which the group with electric light at home had significantly higher light levels during the work week. Figure 6B shows means of light exposure every 15 minutes between the two groups from 17:00 to 19:00 h. In addition, it has been observed a significant difference in activity levels within almost all 15-min intervals from 05:00 to 07:00 h (Mann-Whitney U test; p < 0.05). Figure 7B shows means of activity levels in early morning during the work week.

However, repeated measures ANOVA failed to show significant differences in activity levels and light exposure between the two groups when analyzed together, with day of week (work day and day-off) as a factor (Figures 5AB, 6A and 7A).

## Discussion

Our findings demonstrate a significant impact of availability of electric light at home on the timing of sleep (Phase 1 and 2) and melatonin onset (Phase 2) in workers living in the Amazon. Workers with electric light at home had later sleep onset time (according to subjective and objective measures) during the work week and days-off compared to workers without electric light at home. This finding supports studies showing the delaying effect of electric light exposure in the evening on sleep onset time^17^,^18^.

The results (Phase 1) also showed significantly reduced sleep duration in workers with electric light at home. Although this reduced sleep across the work week may lead to increased sleep debt in this group, no difference in the subjective sleepiness measurements between the two groups was observed. Moreover, in Phase 2 of the study the differences in objective sleep duration between the two groups were not statistically significant. Nevertheless, the questionnaire used for the Phase 1 study, asked a single question about bedtime, thus, the subjects needed to record their “usual/habitual bedtime”. On the other hand, the actigraph recorded sleep onset data across several days (including work days and days off). Minor differences between these measures are to be expected.

To the best of our knowledge, this is the first study that has demonstrated the phase relationship between melatonin onset, sleep timing and the prevailing light/dark cycle in adults living without electric light in a real life situation, i.e having to fulfill their work and social obligations, compared with adults who had electric light at home. To assess the impact of electric light on sleep timing, a unique population (relatively isolated, ethnically homogenous), all performing the same work has been studied.

The rubber tappers without electric light at home showed entrainment to the light-dark cycle similar to that found by Wright *et al.*^16^ in a group of adults living outdoors in camping tents for one week. These authors also observed synchronization between the internal circadian system and solar time after exposure to only natural light.

The detailed analysis from Phase 2 of the study showed that the time of sleep onset, however, was delayed in both groups of rubber tappers during their days-off, irrespective of the presence or absence of electric light. The fact that the time interval between melatonin onset and sleep onset (phase angle) was different on the day-offs in both groups suggests that the endogenous melatonin rhythm, a reliable marker of circadian phase, was entrained to the light-dark cycle whereas sleep onset changed according to work hours. According to Savides *et al.*^19^, natural light exposure for human beings is unpredictable in terms of intensity and time, however, in our study, exposure to the natural light-dark cycle was very stable, since the population lives near the Equator. In addition, most of the rubber tappers have been living in this 12 h light/dark (LD 12/12) environment for their whole life, which may also contribute to their observed entrainment to the natural light-dark cycle. The rubber tappers were also assumed to have similar genetic characteristics, however, further studies are needed to confirm this. The rubber tappers worked outdoors being exposed to natural daylight every day, thus, in our study population the ability of an organism to adapt to a predictable environmental change (such as 12 h light/dark) is being observed, whereas in Savides’s study^19^ participants were not exposed to a stable natural light/dark cycle. During the work week the rubber tappers from both groups woke up before sunrise, and soon after sunrise during their days-off indicating the rubber tappers were entrained to a predictable and unchanging 12 h L/12 h D light-dark cycle. These results corroborate models as proposed by Pittendrigh and Aschoff emphasizing the importance of light-dark transitions at twilight and the phase shifts in response to light every dawn and dusk for circadian entrainment^20^.

The natural light/dark conditions in our study thus promoted synchronization between the environmental light-dark cycle and the circadian system, whereas the early work time may have disrupted this balance during the work week. For those who had electric light at home it seems to be a double-burden to disrupt circadian alignment, the exposure of evening light at home in addition to the early work schedule. In other words, the observed delayed sleep onset in this group might not be compensated with a later awakening due to work hours, although individual differences should be taken into account. The increased activity between 05:00–07:00 h in the no electric group might also be explained by individual differences, since some of the rubber tappers were probably awake at that time. Moreover, a delay in sleep and wake times was observed in the days-off for both groups, suggesting a role of working hours on sleep timing.

An intriguing finding from Phase 2 of our study is the similar time of melatonin onset during both work days and days-off for those who did not have electric light at home. One possible explanation for this consistency in melatonin timing might be exposure to the same dawn signal since the rubber tappers woke up before sunrise during the work week, and just after sunrise during their days-off. Our findings support the study of Danilenko *et al.*^21^ that suggested a full dawn signal characterized by gradual increases of light may aid adaptation of the organism to the day-night cycle. It may be that the complete dawn signal together with the gradual decrease of light during dusk for the whole week maintained entrainment to the light-dark cycle in the group without electric light at home.

In the remote Amazonian area that was studied the availability of electric light included a few light bulbs at home, a refrigerator, a fan and perhaps a sound system/electric radio. The rubber tappers with electric light at home did not have computers, electronic tablets, mobile phones or videogames that might increase the number of available activities in the evening and also lead to a more delayed sleep onset time. Also, drycell lighting technology was not commonly in use within the studied population due to higher costs. Thus, the delay in sleep onset observed in the current study may be related to relatively dim electric light exposure in the evening (approximately 30 lux inside the houses with electric light) and not to modern electronic devices. The differences in spectral composition and colour temperature between electric tungsten and fluorescent bulbs (more blue-enriched, higher colour temperature light) and non-electric light sources (candle, kerosene wick lantern lamps) may explain the effectiveness of the relatively dim light electric on sleep timing.

In support of our findings, studies in controlled laboratory conditions have shown that exposure to a low level of illuminance is sufficient to phase shift and entrain the circadian system^7^,^11^,^21^. It is thus possible that circadian entrainment occurred in this real-life situation where the only source of light was a few electric light bulbs at home. In a laboratory study with highly controlled lighting conditions it has been demonstrated that the phase angle of entrainment changes according to consecutive exposure to bright light^22^. After several days of bright light exposure in the laboratory, the subjects showed an increase in their phase angle (difference between melatonin onset and sleep onset), which is consistent with what we have observed in the rubber tappers with electric light at home.

We have not found many significant differences between the two groups regarding demographic factors on both phases of the study. Data from Phase 1 showed that were significantly more single rubber tappers among those without electric light at home. Having a partner might also have contributed to postponed bedtime for rubber tappers with electric light at home. Nevertheless, there is some evidence showing that sleep concordance may vary according to each couple’s characteristics^23^. As the number of female participants in our studied population was very small, we were not able to investigate differences on sleep between males and females.

Chronotype, as measured by the Munich Chronotype Questionnaire, was also significantly later for those who had electric light at home compared to those with no electricity, at least for the whole sample (Phase 1). In a recent study Nag and Pradhan^24^ showed a higher percentage of morning types in a remote region when comparing diurnal preferences in three different areas, remote (without electric light), rural (with electric light) and urban area (with electric light)^24^. Thus, our study supports the idea that, even in a relatively ethnically homogenous population it is possible to observe differences in chronotype depending upon the availability of electric light. This result, however, could not be observed in Phase 2, probably due to the small sample size in this analysis, which is a limitation of our study.

In short, the current findings suggest maintenance of the synchronization between the circadian timing system (as measured by melatonin timing) and the light-dark cycle in the workers lacking electric light at home, probably due to exposure to both the dawn and dusk light signals. In a natural environment the circadian system may thus be fully synchronized to the light-dark cycle whereas the timing of work and social activities may change sleep times and length.

Our findings also suggest that low intensity artificial light around dusk might disturb the alignment between the circadian system and the natural light/dark cycle as evidenced by a delay in the time of melatonin onset for those with electric light at home, compared to those without electric light. Early work time seems to have a relevant role to disrupt the circadian system during the work week. Put together, these findings will help to understand the impact of work schedules and artificial light exposure in our society on sleep and circadian timing.

## Methods

This study was conducted in two phases. In Phase 1 the participants were interviewed by a trained team from a local University. This phase aimed to collect demographic, life style and sleep data. Phase 2 included a subgroup of rubber tappers that were intensively studied and asked to collect saliva and to wear light sensors (MicroMiniLight Sensor*™,* Ambulatory Monitoring, Inc.) as well as actigraphs (Mini Basic Motionlogger Actigraph*™* Ambulatory Monitoring, Inc.) during the work week and days-off.

### Study phases

#### Phase 1

 

### Study participants

Our study population was rubber tappers who worked on the extraction of latex, which is the raw material for the manufacture of condoms in a local governmental factory. Out of the 398 rubber tappers living in the Chico Mendes Extractive Reserve located in Amazon, Brazil (latitude: 10°39′06″S; longitude: 68°30′16″W), 340 were interviewed (85% of population).

The rubber tappers generally carry out their latex extraction activities from 06:00 to 16:00 h from Monday to Friday. During this time the rubber tappers enter the forest via trails and make incisions in the rubber tree trunks in order to extract the latex rubber which they then collect several hours later.

### Data collection

Data collection for Phase 1 was performed between September and November 2011, when the average for sunrise was 06:20 h (±0.10 SEM), and for sunset was 18:33 h (±0.04 SEM). The participants were interviewed in order to complete a form with questions on sociodemographic characteristics (age, gender, civil status, education level, birthplace, electricity at home), anthropometric measurements (self-reported weight and height to calculate BMI), and, lifestyle (smoking, physical activity, alcohol intake), sleep data (Karolinska Sleep Questionnaire), musculoskeletal pain (Nordic Questionnaire for the Analysis of Muscoloeskeletal Symptoms), chronotype (*Munich Chronotype Questionnaire)*, diurnal preference (*Diurnal preference scale*) and self-reported morbidities. Reference values established by the World Health Organization^25^ were used to classify BMI according to normal/obese. The questionnaires used in this study are described below.

The Nordic Questionnaire for the Analysis of Muscoloeskeletal Symptoms^26^ was applied to evaluate the prevalence of musculoskeletal symptoms during the past 12 months. This is a questionnaire with a body map where nine body parts are shown for the respondent to identify the body parts more easily. Below the body map there is the following question: “Do you have musculoskeletal symptoms in any part of the body during the past 12 months?” Each “yes” for a body part was counted as 1 score, thus the scale ranged from 1 to 9 scores.

The Karolinska Sleep Questionnaire^27^ has three independent indices related to sleep problems. The first index, sleep quality, was derived from four items: difficulties falling asleep, disturbed sleep, repeated awakenings, premature awakening. The second index, awakening problems, comprised of two items: difficulties awakening and not well rested on awakening. The third index, sleepiness, included three items: nodding off at work, nodding off during leisure-time, fighting sleep—an effort to remain awake. The response alternatives were: “always/every day (5)”; “mostly/several days per week (4)”; “sometimes/several times per month (3)”; “seldom/a few times per year (2)”; “never (1)”. The three indices were dichotomized at the value ≥4 (yes, problems) and <4 (no problems).

The Munich Chronotype Questionnaire (MCTQ) asks simple questions in regards to the timing of sleep and wake habits, such as the bedtime and awakening, sleep latency, and sleep inertia for work and for free days separately. On the basis of these parameters, more parameters can be computed, such as mid-sleep, sleep duration, social jetlag^28^.

The Diurnal Preference instrument is a short scale with high internal reliability for use as a measure of diurnal type (morning or evening preference) in different time periods. The score ranges from 7 to 28 points, in which values near seven correspond to a more evening character^29^.

Several strategies were adopted to facilitate access to the rubber tappers since the vast majority of these workers reside in remote locations. Interviews were conducted at the following venues: workers’ homes; collection centers (places where workers deposit the rubber to be collected by workers from the factory); municipal markets, and during itinerant health actions held in remote locations, where the researchers were allowed to accompany the municipal health care team.

#### Phase 2

 

### Inclusion and exclusion criteria

Rubber tappers volunteered participation in Phase 2 of the study. However, those who had a disease or were taking medication that could affect sleep patterns and melatonin production were excluded. Thus, 42 rubber tappers (out of 340, 12.3%) participated in Phase 2. Data collection was performed between May and July, 2012, when sunrise occurred on average at 06:48 h (±0.04 h SEM ) and sunset at 18:24 h (±0.04 h SEM) (data from the National Observatory in Brazil).

### Actigraphs and light sensor

To obtain information on the rest/activity cycle, 42 rubber tappers wore an actigraph *(Mini Basic Motionlogger Actigraph™, Ambulatory Monitoring, Inc)* on the non-dominant wrist for 10 consecutive days. Using validated algorithms, it was possible to establish relationships between the cycle of rest/activity and sleep/wake cycle . Daily sleep diaries were used to complement the actigraphy data by cross-checking sleep onset and offset times. This means the sleep diary data (onset and wake up time) was used to set the time period within which the actigraph algorithm calculates the actigraphic sleep parameters. The amount of light exposure was measured for 10 days by a light sensor (MicroMiniLight Sensor*™*, Ambulatory Monitoring Inc), which was worn as necklace.

Some light sensors did not work due to the humidity in the forest. Thus, the sample size for analyses of sleep timing was 37 rubber tappers (n = 17 with electric light and n = 20 without electric light). The sample size for analyses of rest-activity and light exposure was 20 rubber tappers (n = 7 with electric light and n = 13 without electric light). During the first hours in the morning (05:00–07:00 h) and the crepuscular hours (17:00–19:00 h) 15-min bins have been calculated to analyze in detail activity-rest levels and light exposure, respectively.

### Salivary melatonin (Dim Light Melatonin Onset)

Saliva collections were arranged during one work day (on Friday) followed by one day off (on Saturday) for each participant. These collections were performed in a dim light environment with the subjects remaining seated (facing indoors) on a covered porch surrounded by trees. The light intensity varied at dusk, where the first measure was on average 33 lux and the last one was 0 lux, measured at the participants’ eye level (luxmeter THDL-400, Instrutherm™). Saliva collection was performed every 30 minutes from 16:30 to 20:00 h. The workers were asked to refrain from consuming caffeine-containing beverages and food during the time of collection. The samples were collected in polyester tubes and placed in a cooler box containing ice for subsequent storage (−20^o^ C). The workers remained seated during saliva collection to prevent postural changes from interfering with the melatonin measurement^30^, and subjects wore sunglasses during visits to the restroom.The saliva samples were shipped on dry ice to the University of Surrey, UK where melatonin was assayed using existing radioimmunoassay technology (Stockgrand Ltd.).

To estimate DLMO we calculated the mean baseline melatonin values ±2 x SD of the mean baseline melatonin values (pg/ml) as the onset time and then assume a linear increase between the sampling time points^31^. DLMO could not be calculated in some subjects (either on work days or days off) since there was no clear baseline melatonin with a consistent rise in melatonin concentrations across 2 or more consecutive samples, thus the sample size for melatonin timing was reduced from 42 to 33 workers (n = 13 with electric light, n = 20 without electric light).

### Data analysis

The raw data were inspected for underlying distribution via histograms and examined parity with a Gaussian distribution using the Kolmogorov–Smirnov test. For frequency type data (e.g., sociodemographic characteristics), chi-square and Fisher exact tests were employed, whereas for continuous type data (e.g. age, BMI), comparison of means between the two groups (with electric light versus no electric light) was performed using independent Student’s t-tests or Mann–Whitney U tests (according to data distribution). A repeated measures ANOVA was used to test the main effects and the interaction between day of week (week and weekend) and electric light (with and without electric light) on sleep onset, sleep offset, DLMO, phase angle, activity levels and light exposure. All tests were considered statistically significant when p ≤ 0.05. All data analysis was carried out with Statistica version 7.0 and Stata version 12.0 software packages.

### Ethical issues

Data were collected only after study approval by the Research Ethics Committee of the School of Public Health, University of São Paulo, and the study was carried out in accordance with the ethical standards laid down in the 1964 Declaration of Helsinki and its later amendments. Written informed consent was obtained from all participants.

## Additional Information

**How to cite this article**: Moreno, C. R. C. *et al.* Sleep patterns in Amazon rubber tappers with and without electric light at home. *Sci. Rep.* **5**, 14074; doi: 10.1038/srep14074 (2015).

## Figures and Tables

**Table 1:** Demographic data and reported sleep disturbances from rubber tappers with and without electric light at home in Phase 1 (n = 340) and Phase 2 (n = 42) of the study.

Variables	Categories	**Phase 1**					**Phase 2**				
		**Electric light**		**No electric light**		**p**	**Electric light**		**No electric light**		**p**
		n	(%)	n	(%)		n	(%)	n	(%)	
Gender	Female	8	(8.3)	21	(8.6)		1	(5.9)	0	(0)	
	Male	89	(91.7)	222	(91.4)	0.91^a^	16	(94.1)	25	(100)	0.40^b^
Civil status	With partner	78	(80.4)	170	(70)		16	(94.1)	20	(80)	
	Without partner	19	(19.6)	73	(30)	0.05^a^	1	(5.9)	5	(20)	0.37^b^
Education level	Illiterate	23	(23.7)	70	(28.8)		2	(11.8)	5	(20)	
	Primary school	67	(69.1)	166	(68.3)		12	(70.6)	18	(72)	
	High school	7	(7.2)	7	(2.9)	0.15^a^	3	(17.6)	2	(8)	0.55^b^
Birthplace	Extractive Reserve	79	(81.5)	187	(77)		15	(88.2)	18	(72)	
	Other places in Acre	14	(14.4)	42	(17.3)		2	(11.8)	6	(24)	
	Other states in Brazil	4	(4.1)	14	(5.7)	0.65^a^	0	(0)	1	(4)	0.54^b^
Smoking	No	47	(48.5)	124	(51)		6	(35.3)	12	(48)	
	Yes	50	(51.5)	119	(49)	0.67^a^	11	(64.7)	13	(52)	0.41^a^
cSleep quality problems	No	86	(88.7)	223	(91.8)		15	(88.2)	23	(92)	
	Yes	11	(11.3)	20	(8.2)	0.37^a^	2	(11.8)	2	(8)	0.65^b^
cAwakening problems	No	93	(95.9)	237	(97.5)		16	(94.1)	24	(96)	
	Yes	4	(4.1)	6	(2.5)	0.42^a^	1	(5.9)	1	(4)	0.78^b^
cSleepiness	No	91	(93.8)	215	(88.5)		17	(100)	25	(100)	
	Yes	6	(6.2)	28	(11.5)	0.14^a^	–	–	–	–	–

^a^Chi-square test.

^b^Fisher Exact Test.

^c^Karolinska Sleep Questionnaire.

**Table 2:** Characteristics and life style (means and SEM) of rubber tappers with and without electric light at home in Phase 1 (n = 340) and Phase 2 (n = 42).

Variables	**Phase 1**					**Phase 2**				
	**Electric light**		**No Electric light**		**p**	**Electric light**		**No Electric light**		**p**
	**χ**	**(SEM)**	**χ**	**(SEM)**		**χ**	**(SEM)**	**χ**	**(SEM)**	
Age (years)	40.5	(1.3)	42.6	(0.9)	0.21^a^	38.1	(2.6)	35.7	(2.0)	0.53^b^
BMI (kg/m^2^)	24.0	(0.4)	23.6	(0.2)	0.22^b^	24.1	(0.7)	23.1	(0.7)	0.09^b^
Musculoskeletal pain (score 1–9)	3.3	(0.3)	3.3	(0.2)	0.78^b^	2.8	(0.6)	3.6	(0.4)	0.27^b^
Diurnal preference (score)	24.0	(0.3)	24.4	(0.1)	0.52^b^	22.4	(0.6)	23.6	(0.5)	0.14^a^
Sun exposure – Work days (hours)	5.2	(0.3)	5.4	(0.2)	0.31^b^	4.5	(0.5)	5.2	(0.5)	0.47^a^
Chronotype – (time)	01:00	(1.0 h)	00:36	(0.9 h)	<0.01^a^	01:17	(1.0 h)	00:54	(1.0 h)	0.44^a^

^a^Student’s t-test.

^b^Mann-Whitney.
